# Towards Low Energy Atrial Defibrillation

**DOI:** 10.3390/s150922378

**Published:** 2015-09-03

**Authors:** Philip Walsh, Vivek Kodoth, David McEneaney, Paola Rodrigues, Jose Velasquez, Niall Waterman, Omar Escalona

**Affiliations:** 1 Centre for Advanced Cardiovascular Research, Ulster University, BT37 0QB, UK;E-Mails: Walsh-P3@email.ulster.ac.uk (P.W.); mailto:Rodrigues-P@email.ulster.ac.uk (P.R.); Velasquez-J@email.ulster.ac.uk (J.V.); n.waterman@ulster.ac.uk (N.W.); oj.escalona@ulster.ac.uk (O.E.); 2 The Heart Centre, Royal Victoria Hospital, Belfast, BT12 6BA, UK; E-Mail: Vivek.kodoth@rbch.nhs.uk; 3 Craigavon Area Hospital, Craigavon, BT63 5QQ, UK; E-Mail: David.McEneaney@southerntrust.hscni.net

**Keywords:** wireless, battery-free, implantable, impedance, RF, defibrillator

## Abstract

A wireless powered implantable atrial defibrillator consisting of a battery driven hand-held radio frequency (RF) power transmitter (*ex vivo*) and a passive (battery free) implantable power receiver (*in vivo*) that enables measurement of the intracardiacimpedance (ICI) during internal atrial defibrillation is reported. The architecture is designed to operate in two modes: Cardiac sense mode (power-up, measure the impedance of the cardiac substrate and communicate data to the *ex vivo* power transmitter) and cardiac shock mode (delivery of a synchronised very low tilt rectilinear electrical shock waveform). An initial prototype was implemented and tested. In low-power (sense) mode, >5 W was delivered across a 2.5 cm air-skin gap to facilitate measurement of the impedance of the cardiac substrate. In high-power (shock) mode, >180 W (delivered as a 12 ms monophasic very-low-tilt-rectilinear (M-VLTR) or as a 12 ms biphasic very-low-tilt-rectilinear (B-VLTR) chronosymmetric (6ms/6ms) amplitude asymmetric (negative phase at 50% magnitude) shock was reliably and repeatedly delivered across the same interface; with >47% DC-to-DC (direct current to direct current) power transfer efficiency at a switching frequency of 185 kHz achieved. In an initial trial of the RF architecture developed, 30 patients with AF were randomised to therapy with an RF generated M-VLTR or B-VLTR shock using a step-up voltage protocol (50–300 V). Mean energy for successful cardioversion was 8.51 J ± 3.16 J. Subsequent analysis revealed that all patients who cardioverted exhibited a significant decrease in ICI between the first and third shocks (5.00 Ω (SD(σ) = 1.62 Ω), *p* < 0.01) while spectral analysis across frequency also revealed a significant variation in the impedance-amplitude-spectrum-area (IAMSA) within the same patient group (|∆(IAMSA_S1_-IAMSA_S3_)[1 Hz − 20 kHz] = 20.82 Ω-Hz (SD(σ) = 10.77 Ω-Hz), *p* < 0.01); both trends being absent in all patients that failed to cardiovert. Efficient transcutaneous power transfer and sensing of ICI during cardioversion are evidenced as key to the advancement of low-energy atrial defibrillation.

## 1. Introduction

Atrial fibrillation (AF) is one of the most common cardiac arrhythmias observed in medicine. It is caused by rapid unsynchronised contractions that give rise to “quivering” of the upper atria. This results in a partial loss of cardiac output. Clinically, atrial fibrillation is the breakdown of synchronised electrical activity in the upper chamber of the heart and is diagnosed on an electrocardiogram (ECG) as a small fluctuating baseline undulation of variable magnitude and morphology at a rate of approximately 350–600 beats/min [1]. Fundamentally, atrial fibrillation is associated with deterioration in cardiac function and increased risk of stroke resulting in significant morbidity and mortality. The most recently published data from the Rotterdam study reports a 5.5% prevalence of AF in individuals over the age of 55 years while AF is currently estimated to account for 30%–40% of all hospitalizations due to cardiac arrhythmias. AF is estimated to affect an estimated 4.5 million people in Europe and 3.0 million adults in the United States; with numbers expected to double over the next 25 years and associated treatment costs currently estimated at in excess of 15.7 billion US dollars per annum within Europe alone. The need for improved and more efficacious therapies therefore remains self evident [2,3,4,5].

For AF patients where pharmacological intervention is either deemed inappropriate or has failed, external electrical cardioversion (the delivery of asynchronized direct current shockacross the patient’s chest in an attempt to restore sinus rhythm) is often used. However, high energy shocks of up to 200 J (typically delivered as a monophasic or biphasic electrical impulse measuring up to 2.5 kV for 12 ms in duration) are required for successful transthoracic cardioversion; giving rise to extreme patient discomfort and necessitating heavy sedation or full anesthesia. Moreover, this method often fails; most commonly in large patients where thoracic impedance is high. This has led to the development of synchronised internal atrial defibrillation (more commonly referred to as internal cardioversion), a procedure which employs either a single or pair of electrically conductive catheters to deliver an electrical shock directly between the distal coronary sinus and the right atrial appendage of the heart in an attempt to restore sinus rhythm. Internal cardioversion of atrial fibrillation has been shown to be effective and safe in a number of clinical studies. In particular, it continues to have a role in the treatment of patients who have previously been unsuccessfully treated using direct current transthoracic cardioversion. After internal cardioversion, the long-term outcome in a patient’s refractory to external cardioversion is good; especially if total arrhythmia duration has been brief [6,7]. Moreover, one of the primary advantages of internal cardioversion is that the energy required for a successful outcome (more commonly referred to as the defibrillation threshold) is substantially reduced; typically by up to a factor of ten (to <20 J). However, the fact remains that synchronised internal cardioversion still necessitates patient sedation and is an invasive procedure with all of the attendant risks of tissue damage, infection and the potential to induce other cardiac arrhythmias.

The potential use of implantable devices for patients with AF has therefore received a great deal of attention. In 1995, the first human implant of a stand-alone implantable atrial defibrillator (IAD) took place. The battery-powered device detected AF and delivered R-wave synchronous defibrillation 12 ms biphasic shocks (chronosymmetric 6ms/6ms) at maximum of 300 V to convert AF to sinus rhythm [8,9,10]. However, the use of IAD’s for the treatment of AF has not yet achieved critical acceptance; predominately due to the impact of unit automaticity on the patients quality of life and the lack of patient tolerance to the discomfort produced by high energy shocks [11,12]. Recent publications indicate that the further advancement of internal cardioversion for AF may therefore result from two specific lines of enquiry: (i) optimisation of the defibrillation shock impulse to achieve the lowest energy necessary to successfully cardiovert a patient (less than 1 J could potentially negate the need for patient sedation) and (ii) investigation of passive (battery free) implantable atrial defibrillators that can facilitate AF arrhythmia detection and cardioversion under controlled circumstance in a non-acute care (out-of-hospital) setting [13,14,15,16,17,18,19,20,21,22,23,24,25]. In respect of the optimisation of electrical shock waveforms to achieve a defibrillation threshold of <1 J, transthoracic impedance (TTI) is a key determinant in the success of both atrial and ventricular defibrillation; due to the fact that cardioversion outcome highly correlates to the current vector delivered to the cardiac substrate. Hence, modern external defibrillators first measure the impedance of the cardiac substrate before delivering a precise amount of energy using an impedance compensated biphasic (ICB) shock waveform. In several studies, biphasic and biphasic impedance compensated defibrillators have been found to be more effective than equivalent high energy monophasic devices [14,15,16,17]. However, there remains a paucity of studies examining the correlation of the intracardiac impedance (DC impedance, dynamic impedance and waveform spectral content) during internal atrial defibrillation to clinical outcomes. In addition, recent publications have indicated that multiple low energy intracardiac shocks may give rise to lower cardioversion thresholds [18,19,20,21,22,23] thereby significantly minimising patient discomfort. Yet again, a paucity of studies comparing the efficaciousness of such protocols exists. In respect of the potential for passive (battery free) implantable defibrillator technologies to enable treatment in a non-acute care setting, the concept has several obvious merits; the issue of implant automaticity and inappropriate shocks is resolved (thereby addressing the potential quality of life associated issues previously identified) while the need for repeat surgery to replace batteries over the lifetime of the patient is entirely eliminated. Consequently, low-energy cardioversion using passive implantable defibrillator technology offers the potential for the development of AF treatment modalities and protocols that could be safely delivered in a non-acute (out-of-hospital) care setting; thereby eliminating the need for repeated hospitalisation and significantly reducing the long term cost burden associated with treatment [20,25].

In this paper we propose and demonstrate a wireless powered implantable atrial defibrillator architecture that enables capture of the intracardiac impedance between successive shocks during the internal cardioversion procedure; thereby enabling implementation of impedance compensated internal atrial defibrillation therapies that are optimized to each individual patient and that could potentially be delivered in a non-acute care (out-of-hospital) setting. An initial trial of the RF architecture developed has successfully demonstrated cardioversion at low-energy and also revealed heretofore unreported observations that intracardiac impedance measurements captured during low-energy cardioversion contain markers in both the time and frequency domain that correlate to low-energy cardioversion outcome. Efficient transcutaneous power transfer, wireless powered *in vivo* sensing and measurement of intracardiac impedance during cardioversion are therefore evidenced as key to the advancement of low-energy defibrillation therapies.

## 2. Methods

### 2.1. Experimental Design

**Figure 1:**
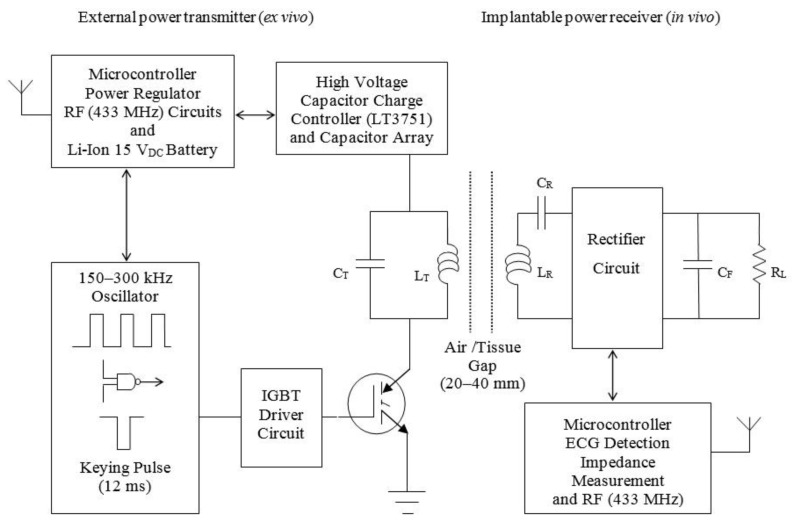
Top-level implantable transcutaneous RF power link architecture.

**Figure 2:**
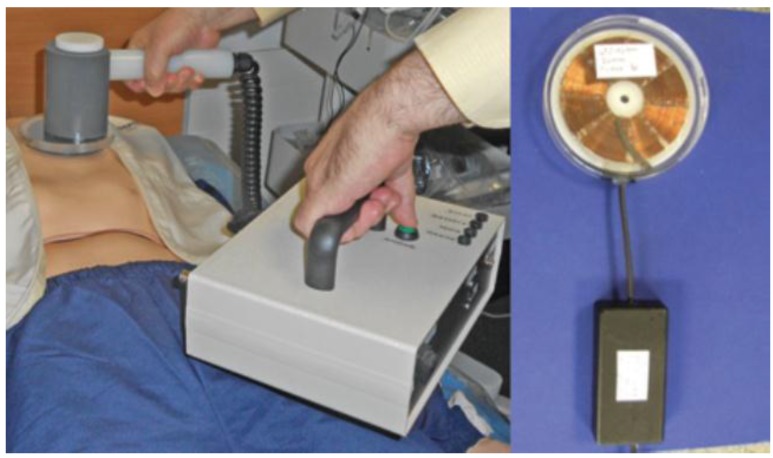
Prototype hand-held (*ex vivo*) radio frequency (RF) power-transmitter and prototype battery-free implantable (*in vivo*) power-receiver (encapsulated for bench characterisation).

Figure 1 shows a top-level schematic of the power transmitter (*ex vivo*) and power receiver (*in vivo*) architecture while Figure 2 provides an image of one of the prototypes developed. The hand-held external power transmitter consists of a microcontroller based unit that contains a tuned parallel RLC resonant tank circuit energized via a capacitor array (three 680 µF 400 V electrolytic capacitors managed by a LT375I charge controller) and driven via synchronous switching of an IGBT (IRG4PH40U) located in the current return path to ground from the primary coil (L_T_ = 9.65 µH: 30 turn spiral coil, inner diameter 20 mm, 0.75 mm insulated copper wire) with resonant capacitor (C_T_ = 10 nF). Similarly, the implantable power receiver consists of a series RLC resonant tank circuit with a primary-secondary turns ratio of 1:1 (L_R_ = 9.65 µH: 30 turn spiral coil, inner diameter 20 mm, 0.75 mm insulated copper wire) and a resonant capacitor (C_R_ = 10 nF) wired in a central tapped configuration that drives a full-wave voltage double circuit; with multiple regulators to provide the range of voltages (6–18 V) required for operation of the integrated control, measurement and communications circuitry. Both circuits (Tx/Rx) were designed for operation at approximately 185 kHz. The output of the receiver coil is then rectified and filtered. Several linear regulators provide the necessary range of voltages (18 V, 12 V and 6 V) required for successful operation of the control, measurement and data communications circuitry on the implant side. The implantable power receiver unit contains a low-power integrated microcontroller (FRDM-KL25ZARM processor) that communicates with the base unit and coordinates between two distinct modes of operation: cardiac sense mode (wake-up, measure the impedance of the cardiac substrate and communicate data to the external base unit) and shock mode (delivery of an ECG synchronised impedance compensated monophasic very-low-tilt-rectilinear shock waveform). In low-power or sense mode, >5 W of power is continuously transferred while receiver temperature monitoring ensures that maximum implant operating temperatures are never exceeded. In this mode, a modified Howland bridge is used to non-destructively determine the impedance spectrum of the cardiac substrate by injecting a sinusoidal test current of fixed magnitude (±98 μA) and measuring the voltage response across a range of frequencies (1–20 kHz). This measurement can then be sent via a dedicated communications link (433 MHz) to the external base unit. This dual-band architecture facilitates independent optimisation of both the power transfer and data-communications links. In the shock delivery mode, an ECG synchronised impedance compensated M-VLTR shock waveform (100 V at 50 Ω for 12 ms) can then be delivered to the cardiac substrate via atrial leads attached to the implanted coil. Several initial prototypes were developed including variants incorporating H-Bridge reversal circuitry to facilitate delivery of monophasic or biphasic shock waveforms.

### 2.2. Simulation and Characterisation

**Figure 3:**
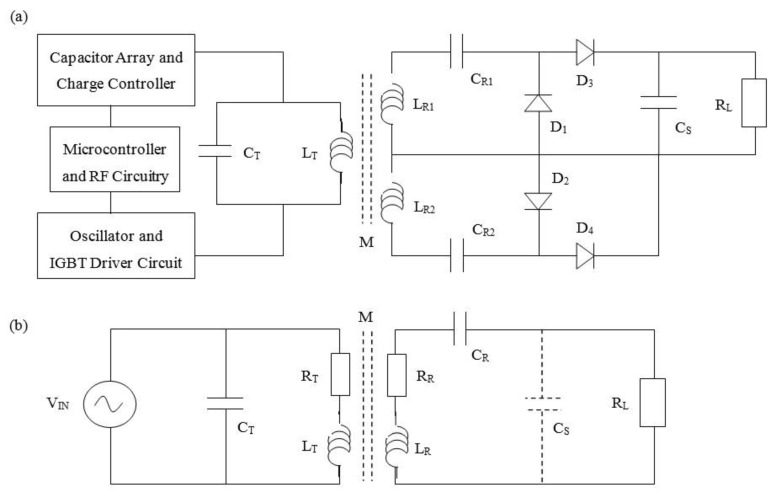
(**a**) Implantable receiver architecture and (**b**) simplified circuit used for analysis.

Theoretical analysis and simulation of the RF architecture developed (Figure 1) was approached as previously reported [24]. Analysis was complicated by the fact that the receiver rectifier circuit effectively takes on a different configuration dependent on the type of waveform being delivered to the patient (monophasic (positive only) or biphasic (with a negative phase component)). In order to approach calculation and estimation of the RF link efficiency, a simplified circuit topology was adopted. Figure 3a shows the actual receiver architecture while Figure 3b shows the simplified model developed; which comprehends the parasitic series resistance for both the transmitter and receiver coils but ignores the associated distributed capacitance.

This parallel-series resonant tank configuration may be modelled as a current-in/current-out topology and effectively provides current and voltage magnification in the transmitter (parallel resonance) and receiver (series resonance), respectively. The impedance of the receiver *Z_RX_* and the transmitter *Z_TX_* circuit can therefore be expressed as:

(1) $$Z_{Rx}=\left(R_{R}+R_{L}\right)+j\left(\omega \cdot L_{R}-\frac{1}{\omega \cdot C_{R}}\right)$$

(2) $$Z_{Tx}=\frac{R_{T}+R_{REF}+j\cdot \omega \cdot L_{T}}{1+\left(R_{T}+R_{REF}\right)j\cdot \omega \cdot C_{T}=\omega^{2}\cdot C_{T}\cdot L_{T}}$$

from which the reflected impedance *Z_REF_* from the receiver to the transmitter circuit may be derived as:

(3) $$Z_{REF}=\frac{\omega^{2}\cdot M^{2}(R_{R}+R_{T})}{{\left(R_{R}+R_{T}\right)}^{2}+{\left(\omega \cdot L_{R}-\frac{1}{\omega \cdot C_{R}}\right)}^{2}}+j[-\frac{\omega^{2}\cdot M^{2}(\omega \cdot L_{R}-\frac{1}{\omega \cdot C_{R}})}{{\left(R_{R}+R_{T}\right)}^{2}+{\left(\omega \cdot L_{R}-\frac{1}{\omega \cdot C_{R}}\right)}^{2}}]$$

where *L_T_* and *L_R_* are the self-inductance values for the transmitter and receiver windings, respectively, *R_T_* and *R_R_* are the parasitic series resistance shown for each coil, respectively, *M* is the mutual inductance between coils, *ω* represents the angular frequency and *R_L_* is the load resistance. Further analysis resolves the receiver and transmitter resonance capacitor values *C_R_* and *C_T_* as:

(4) $$C_{R}=\frac{1}{\omega_{0}^{2}\cdot L_{R}}$$

(5) $$C_{T}=\frac{L_{T}}{\omega^{2}\cdot L_{T}^{2}+\left(R_{T}+R_{REF}\right)}$$

from which the link efficiency (η) is defined as the product between the transmitter (η_T_) and receiver (η_R_) circuit efficiencies where:

(6) $$\eta \left(T\right)=\frac{P_{R}}{P_{TX}}$$

(7) $$\eta \left(R\right)=\frac{P_{L}}{P_{RX}}$$

(8) $$\eta =\;\frac{M^{2}\cdot R_{L}}{\left(R_{R}+R_{L}\right)\left[R_{T}\cdot L_{R}\cdot C_{R}\left(R_{R}+R_{L}\right)+M^{2}\right]}$$

where P_R_ is the power delivered to the receiver circuit, P_TX_ is the total power handled by the transmitter circuit, P_L_ is the power delivered to the load and P_RX_ is the total power handled by the receiver circuit. The self and mutual inductance values adopted and used in the simulation were derived using both Wheeler’s [26] and Grover’s [27] methods. The MATLAB model developed was therefore designed to comprehend: (i) the transmitter and receiver series resistance, the transmitter and receiver self-inductance, (ii) the transmitter-receiver inter-coil inductance, (iii) the impedance of the cardiac substrate (nominally modeled as a 50 Ω resistive load) and (iv) skin and proximity effects based on Terman [28].

### 2.3. Clinical Study

**Figure 4:**
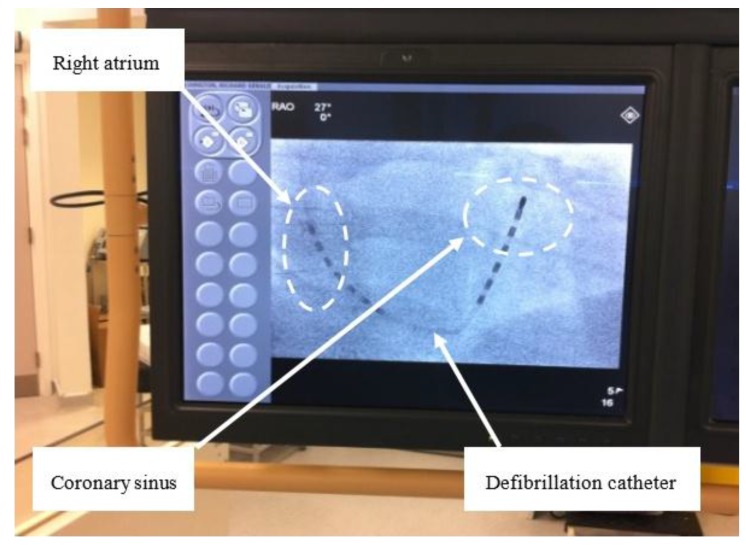
Position of defibrillation leads in the right atrium (RA) and coronary sinus (CS) for internal cardioversion of AF (right anterior oblique view).

In an initial trial of the RF defibrillator architecture developed, 30 patients with persistent AF and with a previous history of failed transthoracic cardioversion were recruited for a study designed to compare the efficacy of RF generated monophasic very-low-tilt-rectilinear *versus* biphasic very-low-tilt-rectilinear shock waveforms [20]. All necessary regulatory and ethical approvals were obtained and all patients who took part provided informed consent. Each patient was fully anticoagulated to achieve an international normalised ratio (INR) of 2 to 3 for ≥4 weeks prior to cardioversion and exclusion criteria were as per current guidelines. The procedures were undertaken in a cardiac catheterisation laboratory. After obtaining venous access, a 6F internal cardioversion catheter was positioned between the distal coronary sinus and right atrial appendage. The defibrillation catheter was then connected to the RF power receiver side of the system. Catheter placement and position was confirmed via fluoroscopy in both right and left anterior oblique views (Figure 4). Intravenous midazolam was administered for adequate sedation prior to commencement of the cardioversion procedure.

**Figure 5:**
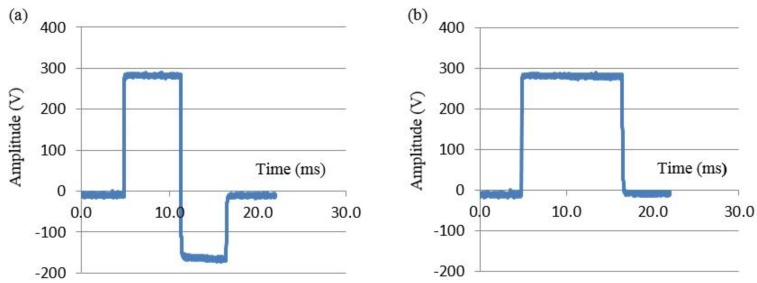
RF defibrillator generated very-low tilt waveforms: (**a**) biphasic (B-VLTR) 12 ms chronosymmetric (6/6 ms) voltage waveform; (**b**) monophasic (M-VLTR) 12 ms waveform.

As part of the study protocol, patients were randomised to therapy with either a biphasic very-low-tilt-rectilinear (B-VLTR) 12 ms chronosymetric (6 ms/6 ms) amplitude asymmetric (negative phase at 50% amplitude) waveform (Figure 5a) or a 12 ms monophasic very-low-tilt-rectilinear (M-VLTR) waveform (Figure 5b) using a voltage step-up protocol of 50 V to 300 V in six steps (Table 1). Patients who failed to achieve sinus rhythm were subsequently crossed over to the opposite arm of the study. To minimise total procedure time, patients that failed to cardiovert for a given shock energy level were progressed in a timely fashion (typically <60 s) to the next shock energy level. Cardioversion success was therefore necessarily defined as the return of sinus rhythm for a period of >30 s.

**Table 1:** Step up protocol with voltage and related shock energy (50 Ω intracardiac impedance assumed).

	Study Arm 1 Biphasic		Study Arm 2 Monophasic	
Step	Voltage	Energy (J)	Voltage	Energy (J)
S1	50	0.38	50	0.60
S2	100	1.50	100	2.40
S3	150	3.38	150	5.40
S4	200	6.00	200	9.60
S5	240	8.62	240	13.80
S6	280	11.75	280	18.81
S7	300	18.50	300	21.60

Post procedure, patient data was retrospectively categorised into four groups according to the type of shock waveform used and outcome achieved: Group-I-successful cardioversion with B-VLTR, Group-II-failed cardioversion with B-VLTR, Group-III-successful cardioversion using M-VLTR, and Group- IV-those who failed to cardiovert with M-VLTR. The primary end point was successful return to sinus rhythm. Associated outcome parameters analysed were leading phase voltage (V), current (I), energy (E), intracardiac impedance (ICI) magnitude (Z(t)), ICI variation between successive shocks (∆Z_T_), ICI magnitude across frequency (Z(f)), ICI spectro-temporal variations between successive shocks (∆Z(f)_T_) and impedance-amplitude-spectrum-area (IAMSA) across frequency.

#### Time and Frequency Domain Signal Processing

For time domain analysis dynamic variation in ICI between successive shocks (∆Z_T_) was investigated. For frequency domain analysis, the spectral content of ICI was derived using a Fast Fourier Transform (FFT) algorithm. Data pairs from the recorded voltage and current (as simultaneously captured during cardioversion) were used to derive ICI as a function of time, Z(t), for the first 4ms of each shock pulse waveform (excluding the rising edge for both waveform types):

(9) $$Z\left(t\right)\;=\;V_{n}/\;I_{n}\left(\text{for n }=\;0,1,2,\;....\text{ N}\right)$$

where *Vn* and *In* are the instantaneous voltage and current values sampled over time, respectively. To minimise spectral leakage a Hanning windowing (µ(t)) was applied:

(10) $$Z\left(t\right)H=Z\left(t\right)\times µ\left(t\right)=\;Z\left(t\right)\times \frac{1}{2}\left(1-\text{cos}\left(\frac{2\pi t}{T}\right)\right)$$

where t, T represent the time and duration of the processed Z(t) signal segments (4 ms), respectively. Subsequently, an FFT was performed to determine the frequency domain function of ICI spectral magnitude components, Z(f), across frequency:

(11) $$FFT\left(k∆f\right)=\;Z\left(f\right)=\;\overset{N-1}{\underset{n=0}{\sum }}f\left(n∆t\right)e^{-i\left(2\pi k∆f\right)\left(n∆t\right)}\left(\text{for k }=\;0,1,2,\;...\text{ N}-1\right)$$

where ∆t = T/N, f_S_ = 1/∆t = N/T, ∆f = 1/T, N is the total number of discrete points sampled, T represents the total sampling period, ∆t is the time interval between data points, f_S_ represents the sampling frequency and ∆f is the frequency resolution of the resulting spectral plot.

The sampling frequency was 500 kHz and the Nyquist criterion dictated that the upper frequency of the spectral response characteristic examined was therefore limited to 250 kHz. Based on a 4096 point FFT, a 4 ms sample yielded a spectral resolution of approximately 122 Hz. The magnitude spectral function of ICI (Z(f)) from 0–20 kHz was derived using both MS-Excel Data Analysis Package and the MATLAB signal processing platform, while the spectro-temporal variation of ICI within the selected frequency range and between successive electrical shocks (∆Z(f)_T_) was calculated in units of ohms and impedance-amplitude-spectrum-area values (IAMSA) was calculated in units of ohms-Hz (Ω-Hz).

## 3. Results and Discussion

### 3.1. Simulation and Experimental Characterisation

Numerical simulations of the proposed RF transmitter-receiver architecture were undertaken as previously reported; using MATLAB and PSpice to evaluate several possible prototype coils for the application proposed [24]. Table 2 presents the self and mutual inductance values derived and used in the simulation for wire diameters ranging from 0.4 to 0.85 mm and also contains the calculated transcutaneous link efficiencies for an inter-coil separation of 25 mm with resonant frequencies from 208 to 212 kHz both with and without the modeling of alternating current effects.

**Table 2:** Self and mutual inductance values derived using Wheeler’s [26] and Grover’s [27] methods for wire diameters ranging from 0.40 to 0.85 mm (Tx = Rx coil diameter) and calculated transcutaneous link efficiency for an inter-coil separation of 25 mm with resonant frequencies ranging from 208 to 212 kHz both with and without the modelling of alternating current effects.

Wire Diameter (mm)	Self-Inductance (µH) [Wheeler's Method]		Self-Inductance (µH) [Grover's Method]		Mutual-Inductance (µH) [Grover's Method]
0.75 0.85 0.40	39.65 38.32 34.89		39.08 38.20 34.43		3.52 2.93 1.29
**Resonant Frequency (kHz)**	**Tx/Rx Wire Diameter (mm)**	**Tx/Rx Resonant Capacitor (nF)**		**Estimated Link Efficiency** **with and without alternating current effects**	
208 211 212	0.85 0.75 0.40	17.40/17.41 14.89/13.57 16.38/16.38		0.76/0.63 0.65/0.54 0.12/0.10	

**Figure 6:**
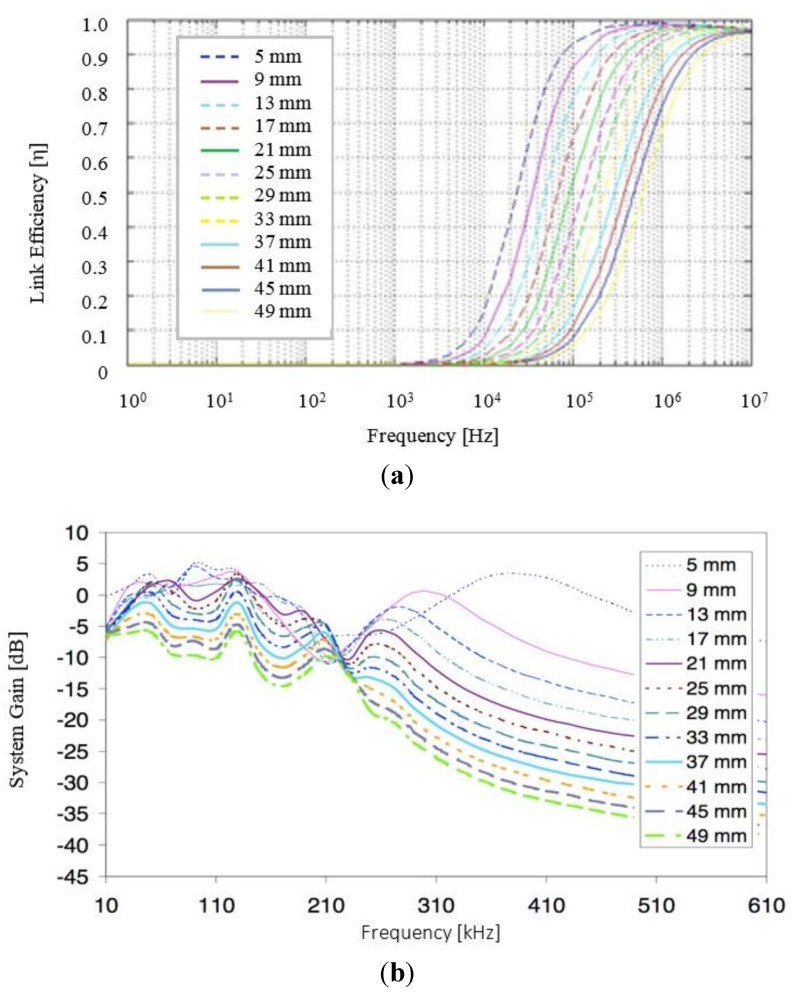

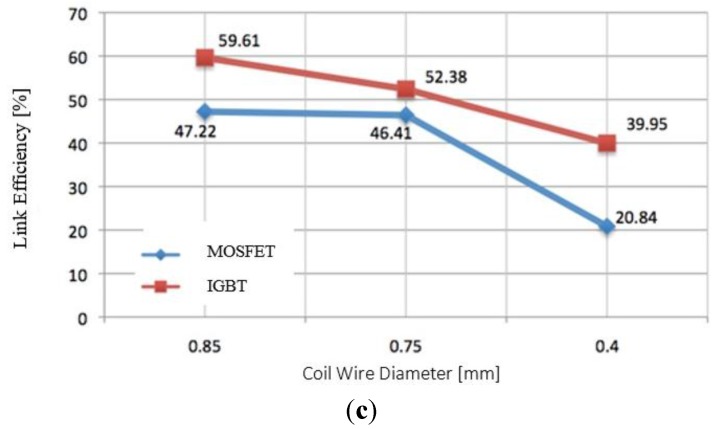
(**a**) Link efficiency *versus* resonant frequency as a function of inter-coil separation; (**b**) gain-frequency characteristics *versus* resonant frequency and (**c**) measured link efficiency *versus* wire diameter at operating resonant frequency of 185 kHz with both an IGBT and a MOSFET for switching.

The overall efficiency and stability of the RF power link in development was also modeled as a function of inter-coil separation. With reference to Figure 6a which plots the overall link efficiency *versus* resonant frequency for a range of inter-coil separations (5–49 mm), as expected the link efficiency display a typical “S” curve and is seen to be heavily dependent on the aforementioned parameter; where for a resonant frequency of 185 kHz an overall transfer efficiency of approximately 60% is theoretically expected. Similarly, Figure 6b plots the gain-frequency characteristics *versus* resonant frequency for the entire system; where again it is evident that an overall gain of approximately −3 dB (50% power, 70% voltage) is achieved for an inter-coil separation of approximately 25 mm at the proposed frequency of operation (185 kHz). For comparative purposes, Figure 6c plots the actual link efficiency measured during testing of a number of experimental prototype coils *versus* wire diameter at an operating frequency of 185 kHz with both an IGBT (IRG4PH40U) and a MOSFET (IFRP260N) examined for potential use as the transmitter resonant tank switching device and with an approximate 52% efficiency for a wire diameter of 0.75 mm achieved.

**Figure 7:**
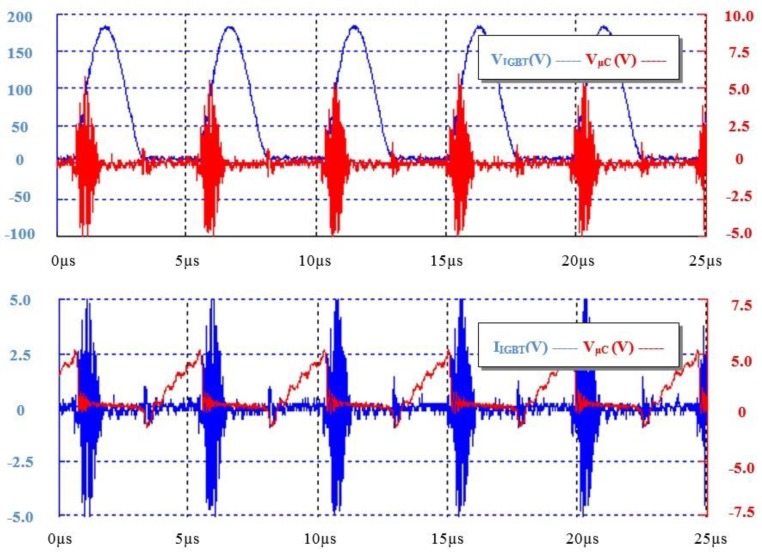
System characterisation data—TX microcontroller (VµC) control signal *versus* IGBT transistor switching voltage (V_IGBT_) and current (I_IGBT_).

Based on the preliminary simulations and experiential work undertaken, architecture sub-systems per Figure 1 were prototyped and fully characterized under laboratory conditions. On the transmission side, Figure 7 shows oscilloscope plots of the control signal from the microcontroller driving the power transmission resonant tank and the voltage and current waveforms that developed; where it is readily observed that at a switching frequency of approximately 185 kHz (T = 5.4 µs) the control signal (0–5 V) modulates the tank voltage and current from 0–180 V at 0–2.5 A.

**Figure 8:**
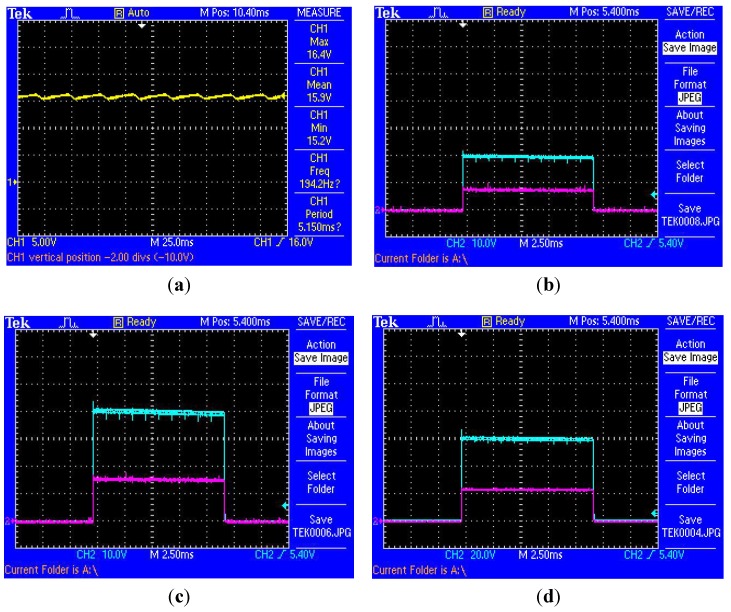

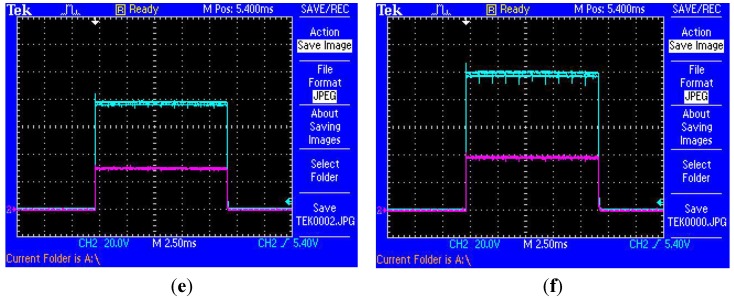
Implant side rectified and regulated output voltage and current for inter-coil separation of 25 mm with R_LOAD_ = 50 Ω at f~185 kHz: (**a**) in sense mode 15.9 V (5 V/div) at 0.32 A (5.1 W); with <8% ripple, Tx-Sense Mode = 15 V, Y-Axis: Output voltage (yellow) = 15.9 V / 0.32 A, (5 V/div); X-Axis: Timebase = 250 ms (25 ms/div); (**b**) Tx-Shock Mode Voltage Setting = 20 V, Y-Axis Ch-1 (green): Output voltage = 19.6 V (10 V/div), Y-Axis Ch-2 (red): Output current = 0.36 A (0.5 A/div); (**c**) Tx-Shock Mode Voltage Setting = 40 V, Y-Axis Ch-1 (green): Output voltage = 39.6 V (10 V/div), Y-Axis Ch-2 (red): Output current = 0.74 A (0.5 A/div); (**d**) Tx-Shock Mode Voltage Setting = 60 V, Y-Axis Ch-1 (green): Output voltage = 59.6 V (20 V/div), Y-Axis Ch-2 (red): Output current = 1.12 A (0.5 A/div); (**e**) Tx-Shock Mode Voltage Setting = 80 V, Y-Axis Ch-1 (green): Output voltage = 76.8 V (20 V/div), Y-Axis Ch-2 (red): Output current = 1.48 A (1 A/div); (**f**) Tx-Shock Mode Voltage Setting = 100 V, Y-Axis Ch-1 (green): Output voltage = 98.4 V (20 V/div), Y-Axis Ch-2 (red): Output current = 1.9 A (1 A/div); with an average waveform tilt of <3.5% measured.

On the receiver side, Figure 8a shows the oscilloscope plot taken from the implant side rectified and regulated output voltagefor a load resistance (R_LOAD_) = 50 Ω at a frequency of 185 kHz. With reference to Figure 8a, in sense mode, V_RX_ = 15.9 V at 0.36 A (5.1 W continuous) with <8% ripple achieved. With reference to Figure 8b through Figure 8f, in shock mode, oscilloscope plots were taken from the implant side (rectified and regulated output voltage) for Tx settings of 20 V, 40 V, 60 V and 100 V, respectively; with a waveform tilt of <3.5% observed. Over extended testing an overall power transfer efficiency across a 25 mm air-skin gap of approximately 47% (worst case) was achieved. Bench characterisation data was therefore found to be in good agreement with both numerical calculations and simulation.

### 3.2. Clinical Study Outcomes and Results

As previously stated, in an initial trial of the RF defibrillator architecture developed 30 patients with persistent AF and with a previous history of failed transthoracic cardioversion were recruited for a study designed to compare the efficacy of RF generated monophonic very-low-tilt-rectilinear *versus* biphasic very-low-tilt-rectilinear shock waveforms; with the baseline characteristics of the 30 patients randomnised in the two arms of the study as previously reported [20]. Seven out of fifteen patients (46%) cardioverted to sinus rhythm with the B-VLTR protocol and 1 out of 15 patients (6%) with the M-VLTR protocol (P = 0.035). When the outcomes of six crossover patients were taken into account, 14 patients in total (46%) were restored to sinus rhythm. For the patients who were successfully treated, mean energy and intracardiac impedance were 8.51 J ± 3.16 J and 73.92 Ω ± 12.01 Ω, respectively.

#### 3.2.1. Quantitative Time Domain Findings

For the time domain analysis, dynamic variations of ICI between successive electrical shocks (∆Z_T_) was analyzed for statistical significance using a student *t*-test for all groups within the study (Table 3). 

**Table 3:** Dynamic changes in ICI(Z_AV_) between shocks measured for the B-VLTR and M-VLTR.

Shock Waveform	Group-I B-VLTR Success (N=7)	Group-II B-VLTR Fail (N=8)	Group-III M-VLTR Success (N=7)	Group-IV M-VLTR Fail (N=8)
Z_AV_ @S1 (Ω ± SD(σ))	79.20 ± 9.16	71.00 ± 17.55	80.50 ± 10.34	74.92 ± 22.68
Z_AV_@ S2 (Ω ± SD(σ))	76.43 ± 10.02	69.28 ± 15.02	78.22 ± 10.16	68.02 ± 22.76
Z_AV_ @ S3(Ω ± SD(σ))	74.25 ± 8.59	67.70 ± 12.95	75.50 ± 9.43	66.80 ± 21.68
|SS − ∆Z_T_|: S1 -> S3 (Ω ± SD(σ)),(*p*-value)	4.95 ± 2.71 *p* < 0.009	3.30 ± 5.63 *p* > 0.154	5.00 ± 1.62 *p* < 0.003	8.12 ± 6.61 *p* > 0.051
Z_AV_ @ S4 (Ω ± SD(σ))	74.14 ± 7.84	67.68 ± 13.32	75.54 ± 9.80	65.88 ± 20.07
|SS - ∆Z_AV_|: S3 -> S4 (Ω ± SD(σ)), (*p*-value)	0.11 ± 2.04 *p* > 0.147	0.02 ± 1.61 *p* > 0.891	−0.04 ± 2.80 *p* > 0.952	0.92 ± 3.40 *p* > 0.404
Z_AV_ @ S5 (Ω ± SD(σ))	71.92 ± 6.46	68.86 ± 12.80	74.51 ± 9.69	66.00 ± 21.00
|SS - ∆Z_AV_|: S4 -> S5 (Ω ± SD(σ), (*p*-value)	2.22 ± 1.61 *p* > 0.100	−1.18 ± 3.50 *p* > 0.254	1.03 ± 3.20 *p* > 0.264	−0.12 ± 3.35 *p* > 0.775

As is self evident, all patients who cardioverted (for both the B-VLTR and M-VTLR treatment protocols) exhibited a significant decrease in ICI (Z_AV_) between the first and third shock (Group I, III: SS−∆Z_T_(S1→S3) = 4.95 Ω (SD(σ) = 2.71 Ω), *p* < 0.01 and SS−∆Z_T_(S1→S3) = 5.00 Ω (SD(σ) = 1.62 Ω), *p* < 0.003, respectively). However, a significant decrease in ICI between successive shocks was absent in all patients who failed to cardiovert (Group II, IV: SS−∆Z_T_ (S1→S3) = 3.30 Ω (SD(σ) = 5.63 Ω), *p* > 0.15 and SS−∆Z_T_(S1→S3) = 8.12 Ω (SD(σ) = 6.61 Ω), *p* > 0.05, respectively). Note that due to sample size for the latter pair of groups, SD(σ) values are relatively large with respect to the mean values rendering meaningless any attempt to assess ICI variation tendencies for SS−∆Z_T_(S1→S3) withineither of thegroups that failed to cardiovert [19].

#### 3.2.2. Quantitative Frequency Domain Findings

**Figure 9:**
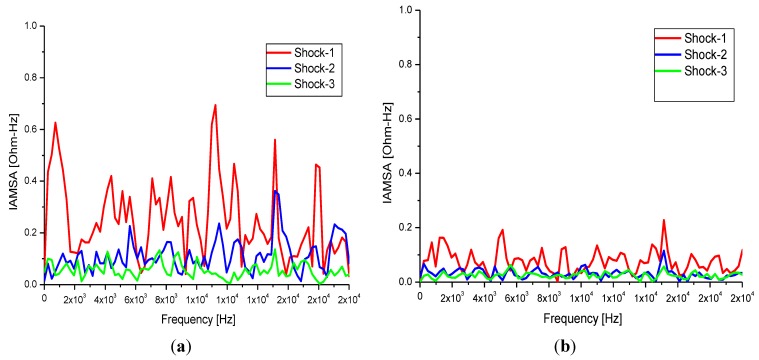

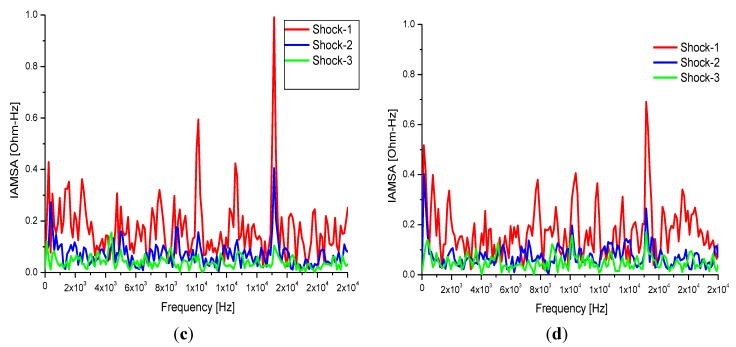
FFT computed impedance magnitude (Ω) spectra for B-VLTR and M-VLTR shocks in the 0–20 kHz frequency range for the first three shocks (S1 -> S3) delivered to patients in Groups-I to IV; (**a**) patient PAF12, B-VLTR Impedance Amplitude Spectrum Area, (Ω-Hz): Group-I B-VLTR Success (0–20 kHz); (**b**) patient PAF18, B-VLTR Impedance Amplitude Spectrum Area, (Ω-Hz): Group-II B-VLTR Fail (0–20 kHz); (**c**) patient PAF14, M-VLTR Impedance Amplitude Spectrum Area, (Ω-Hz): Group-IIIM-VLTR Success (0–20 kHz); (**d**) patient PAF20, M-VLTR Impedance Amplitude Spectrum Area, (Ω-Hz): Group-IV M-VLTR Fail (0–20 kHz).

For frequency domain analysis, an FFT algorithm was used to derive the magnitude of the intracardiac impedance spectral content, Z(f) in ohms (Ω), from 0–20 kHz while dynamic variation in spectral content between successive electrical shocks delivered was quantified by the spectro-temporal parameter ∆Z(f)_T_ and examined for statistical significance using a student *t*-test for all groups within the study. The impedance spectra for a representative patient case in each of the four clinical groups are presented in Figure 9a through Figure 9d.

Similar to what was observed in the time domain, frequency analysis of ICI time series, Z(t), revealed a relative decrease in the impedance magnitude of spectral content of the ICI from 1–20 kHz in all patients who successfully cardioverted. In addition, spectro-temporal variation in ICI between successive shocks delivered, parameter ∆Z(f)_T_, was also quantified in all patients cases from each outcome group. For this analysis, results were summarised as difference values of the impedance-amplitude-spectrum-area (IAMSA) between shock-1 (S1) and shock-3 (S3) (ΔIAMSA (S1-S3)) across various frequency ranges (Table 4).

**Table 4:** ICI spectro-temporal variations of IAMSA for frequency bands and outcome groups.

Group/IAMSA Value (Ω-Hz)	Group-I and III (n = 15(Patients)) B-VLTR & M-VTLR Success		Group-II and IV (n = 15 (Patients)) B-VLTR & M-VTLR Fail	
Shock	S1	S3	S1	S3
IAMSA(f_RANGE-1_)1 Hz–1 kHz (Ω-Hz)	104.42	95.47	85.04	79.99
IAMSA(f_RANGE-2_)1 kHz–10 kHz (Ω-Hz)	8.25	2.98	4.91	1.62
IAMSA(f_RANGE-3_)10 kHz–20 kHz (Ω-Hz)	9.25	2.65	6.29	1.71
IAMSA(1 Hz–20 kHz) (Ω-Hz)	121.92	101.10	96.24	83.32
ΔIAMSA: S1-S3 1 Hz–20 kHz (Ω-Hz ± SD(σ), *p* value)	20.82 ± 10.77 *p* < 0.002 (n = 45 (waveforms))		12.92 ± 10.47 *p*< 0.030 (n = 45 (waveforms))	

Again, as is self evident, for both B-VLTR and M-VLTR treatment protocols, all patients who successfully cardioverted exhibited a significant (*p* < 0.01) decrease in IAMSA value measured atshock-1 (S1) and shock-3 (S3); ∆IAMSA(S1-S3) within the 1 Hz to 20 kHz frequency band (|∆(IAMSA_S1_-IAMSA_S3_)[1 Hz – 20 kHz] = 20.82 Ω-Hz (SD(σ) = 10.77 Ω-Hz), *p* < 0.002) *versus* those who failed to cardiovert (|∆(IAMSA_S1_-IAMSA_S3_)[1 Hz–20 kHz] = 12.92 Ω-Hz (SD(σ) = 10.47 Ω-Hz), *p* < 0.03). These heretofore unreported results are therefore indicative of prognostic attributes in both the time and frequency domain thatcorrelate to cardioversion outcome.

## 4. Discussion

### 4.1. Experimental Findings

In this paper we propose and demonstrate a wireless powered implantable atrial defibrillator consisting of a battery powered hand-held radio frequency (RF) power transmitter (*ex vivo*) and a passive (battery free) implantable power-receiver (*in vivo*) that enables measurement of the intracardiac impedance (ICI) during internal atrial defibrillation. The architecture is designed to operate in two modes: cardiac sense mode (power-up, measure the impedance of the cardiac substrate and communicate data to the *ex vivo* power transmitter) and cardiac shock mode (delivery of a synchronised very-low-tilt-rectilinear electrical shock waveform). An initial prototype was implemented and tested. In low-power (sense) mode, >5 W was delivered across a 2.5 cm air-skin gap to facilitate measurement of the impedance of the cardiac substrate. In high-power (shock) mode, >180 W (delivered as a 12 ms monophasic very-low-tilt-rectilinear (M-VLTR) or as a 12 ms biphasic very-low-tilt-rectilinear (B-VLTR) chronosymmetric (6ms/6ms) amplitude asymmetric (negative phase at 50% magnitude) shock was reliably and repeatedly be delivered across the same interface; with >47% DC-to-DC power transfer at a switching frequency of 185 kHz achieved. In an initial trial of the RF architecture developed, 30 patients with AF were randomised to therapy with an RF generated M-VLTR or B-VLTR waveform using a step-up voltage protocol (50–300 V). Mean energy for successful cardioversion was 8.51 J ± 3.16 J. Subsequent analysis revealed that all patients who cardioverted exhibited a significant decrease in ICI between the first and third shocks (5.00 Ω (SD(σ) = 1.62 Ω), *p* < 0.01) while spectral analysis across frequency also revealed a significant variation in the impedance-amplitude-spectrum-area (IAMSA) within the same patient group (|∆(IAMSA_S1_-IAMSA_S3_)[1 Hz–20 kHz] = 20.82 Ω-Hz (SD(σ) = 10.77 Ω-Hz), *p* < 0.01); both trends being absent in all patients that failed to cardiovert.

### 4.2. Findings Appraisal in View of Previous Reports

Transcutaneous Energy Transfer (TETs) or Inductive Power Transfer (IPT) technology has been widely investigated as a potential solution for wireless energy transfer from *ex vivo* to *in vivo* implants. However, a commercially viable high power TET or IPT system remains a challenging goal; predominately due to the localized heating of tissue that inevitably results from power losses in the receiver coil [29,30,31,32]. Recent publications have reported high power TET or IPT transcutaneous energy transfer efficiencies of up to 93% [29,30,31,32,33,34,35] while the transfer efficiency of our current implementation is closer to an average of 50% across all modes of operation. Significant additional work is therefore now required to model and optimise the coil winding configurations, misalignment tolerance, microelectronic control circuitry and rectifilter associated losses; to achieve improved energy transfer efficiency characteristics. Furthermore, although the power transfer requirements for successful cardioversion appear quite large (several hundred watts), the inherent pulsatile nature of the cardioversion shock waveform used (maximum 12 ms duration) effectively means that maintenance of local temperature rise (over baseline temperature) to <1° (in proximity to the receiver implant and surrounding tissue) is less problematic than in applications necessitating continuous mode operation [33,34,35]. Specifically, in the present design, continuous power is only applied for a very limited time during sense mode operation (typically only a few seconds is required to complete the ‘sense’ cycle) and hence the possibility of raising the skin temperature above 42 ° (thereby resulting in discomfort or skin damage) has been effectively mitigated. However, significant work remains on-going to develop a feedback control loop to automate this aspect of the design and to ensure that losses within the local tissue are within acceptable limits [29,30,31,32,33,34,35].

In parallel, considerable effort has also been devoted by various research teams to optimisation of electrical shock waveforms for internal cardioversion of AF. In particular, AF related clinical studies conducted at the Royal Victoria Hospital Belfast, have shown that by reducing the tilt of the cardioversion waveform the success and efficacy of the shock can be improved [15]. In 2008, Glover *et al.* compared the clinical efficacy of radio frequency (RF) generated low-tilt biphasic shock waveforms (RF B-VLTR) with standard capacitive discharge based biphasic waveforms in patients with induced AF during electrophysiological studies and in patients who failed to cardiovert with transthoracic cardioversion; thereby demonstrating the B-VTLR waveform to be more efficacious with a lower peak voltage required to successfully cardiovert *versus* a conventional biphasic capacitive discharge based waveform reported [16]. More recently, in 2011, Fenton *et al.* published a LEAP (low-energy antifibrillation pacing) protocol and demonstrated the successful termination of AF using sequenced multiple low-energy shocks; reporting that time based delivery of a train of low energy pulses could be used to effectively lower the cardioversion threshold to approximately 13% of the energy normally required for a single shock impulse with a 93% success rate in canine models [22,23]. In this work, a mean internal cardioversion threshold of 8.51 J ± 3.16 J was achieved using a voltage step-up protocol; less than half the current energy recommended for single shock internal cardioversion (20 J). Hence, the physiological mechanisms postulated by Fenton *et al.* as being potentially responsible for the enhanced efficacy of LEAP (shock sequencing) type protocols are thought likely to underpin the results observed; however, extensive theoretical and modeling will also be required to begin to understand the potential advantages of sequenced and step-up defibrillation protocols and the basis for dynamic impedance variation as a reliable correlated marker of favorable conditions for internal cardioversion success. Furthermore, in this particular clinical study (which was based upon a sequenced step-up voltage/energy protocol) success was necessarily defined as the return of sinus rhythm for a period of >30 s to facilitate the rapid progression of patients (that failed to cardiovert when administered a given shock energy level) to the next energy level. However, it is self evident that further investigation of the time based evolution of the spectral content in the 1 Hz–20 kHz range (α (inter-cellular) and β (intra-cellular) dispersion regions) and correlation to long term clinical outcome (>6 months) will also be required to fully understand both the short and long termaspects of the complex electro-mechanical and biological physiological mechanisms at play.

The outcomes to this study are therefore in broad agreement with previous studies and the advancements presented are illustrative of how wireless powered implantable devices and sensors capable of capturing novel electrophysiological measurements can facilitate the development of a better understanding and ultimately more efficient low-energy atrial defibrillation devices and therapies. Efficient transcutaneous power transfer and sensing of ICI during internal cardioversion are therefore evidenced as key to the advancement of low-energy atrial defibrillation.

## 5. Conclusions

In this paper we proposed, developed and demonstrated a wireless powered implantable atrial defibrillator architecture that facilitates measurement of intracardiac impedance during cardioversion. An initial trial of the RF architecture developed achieved a low-energy cardioversion threshold of 8.51 J ± 3.16 J; less than half the current energy recommended for single shock internal cardioversion (20 J). In addition, intracardiac impedance measurements sensed during low-energy cardioversion were found to contain attributes in both the time and frequency domain that correlate to cardioversion outcome. Efficient transcutaneous power transfer, wireless powered *in vivo* sensing and measurement of intracardiac impedance during cardioversion are therefore evidenced as key to the advancement of low-energy defibrillation therapies. Further development of the system and preparations for additional trials focusing on cardioversion waveform optimisation via capture and advanced analysis of the dynamic impedance spectrum of the cardiac substrate across frequency for multi-shock protocols and in larger patient cohorts remain on-going.
